# Mindfulness and pharmacological prophylaxis after withdrawal from medication overuse in patients with Chronic Migraine: an effectiveness trial with a one-year follow-up

**DOI:** 10.1186/s10194-017-0728-z

**Published:** 2017-02-04

**Authors:** Licia Grazzi, Emanuela Sansone, Alberto Raggi, Domenico D’Amico, Andrea De Giorgio, Matilde Leonardi, Laura De Torres, Francisco Salgado-García, Frank Andrasik

**Affiliations:** 1 Neurological Institute “C. Besta” IRCCS Foundation, Headache and Neuroalgology Unit, Via Celoria 11, 20133 Milan, Italy; 2 Neurological Institute “C. Besta” IRCCS Foundation, Neurology, Public Health and Disability Unit, Milan, Italy; 3 eCampus University, Faculty of Psychology, Novedrate, Italy; 4 Department of Psychology, Univeristy of Memphis, Memphis, TN USA

**Keywords:** Chronic migraine, Medication overuse, Pharmacological prophylaxis, Mindfulness

## Abstract

**Background:**

Chronic Migraine (CM) is a disabling condition, worsened when associated with Medication Overuse (MO). Mindfulness is an emerging technique, effective in different pain conditions, but it has yet to be explored for CM-MO. We report the results of a study assessing a one-year course of patients’ status, with the hypothesis that the effectiveness of a mindfulness-based approach would be similar to that of conventional prophylactic treatments.

**Methods:**

Patients with CM-MO (code 1.3 and 8.2 of the International Classification of Headache Disorders-3Beta) completed a withdrawal program in a day hospital setting. After withdrawal, patients were either treated with Prophylactic Medications (Med-Group), or participated in a Mindfulness-based Training (MT-Group). MT consisted of 6 weekly sessions of guided mindfulness, with patients invited to practice 7–10 min per day. Headache diaries, the headache impact test (HIT-6), the migraine disability assessment (MIDAS), state and trait anxiety (STAI Y1-Y2), and the Beck Depression Inventory (BDI) were administered before withdrawal and at each follow-up (3, 6, 12 after withdrawal) to patients from both groups. Outcome variables were analyzed in separate two-way mixed ANOVAs (Group: Mindfulness vs. Pharmacology x Time: Baseline, 3-, 6-, vs. 12-month follow-up).

**Results:**

A total of 44 patients participated in the study, with the average age being 44.5, average headache frequency/month was 20.5, and average monthly medication intake was 18.4 pills. Data revealed a similar improvement over time in both groups for Headache Frequency (approximately 6–8 days reduction), use of Medication (approximately 7 intakes reduction), MIDAS, HIT-6 (but only for the MED-Group), and BDI; no changes on state and trait anxiety were found. Both groups revealed significant and equivalent improvement with respect to what has become a classical endpoint in this area of research, i.e. 50% or more reduction of headaches compared to baseline, and the majority of patients in each condition no longer satisfied current criteria for CM.

**Conclusions:**

Taken as a whole, our results suggest that the longitudinal course of patients in the MT-Group, that were not prescribed medical prophylaxis, was substantially similar to that of patients who were administered medical prophylaxis.

## Background

Headache disorders are common disabling conditions that, in the last Global Burden of Disease study, were rated as the sixth cause of disability [1]. Further, medication overuse headache was rated as the 18^th^ and, among those aged under 50, migraine was rated as the third cause of disability [2]. Chronic Migraine (CM), is construed as a negative evolution of episodic migraine, based on the findings that approximately 2.5% of episodic migraineurs progress to CM each year [3], with a prevalence of approximately 2% [3, 4]. CM is diagnosed when headache episodes occur more than 15 days/month (with at least 8 displaying migraine headache features) for more than three months [5], and is frequently associated with overuse of acute medications. This has been hypothesized to be one of the chief factors contributing to migraine chronification [6, 7] and, when overuse of medicine reaches a level to warrant a diagnosis of Medication Overuse (MO), it further complicates CM making it particularly difficult to manage. Chronic Migraine associated with Medication Overuse (CM-MO) is diagnosed when the intake of headache medications for headache episodes is greater than 15 days/month for simple analgesics, or exceeds 10 days/month for triptans, opioids, ergotamine or combinations of certain drugs [5]. A recent review of the literature evidenced that analgesics and opioids are associated with a higher risk of developing MO and the authors concluded that the so-called “migraine-specific” treatments, namely triptans and ergots, should be preferred as they are less frequently associated with development of overuse and disease chronification [8].

Patients with CM-MO present therapeutic challenges and require multidisciplinary care, including pharmacological and non-pharmacological therapeutic approaches [9]. Various pharmacological therapies have more recently been developed to help these patients better manage their condition [10–13], but symptom resolution is not always optimal and up to one third of patients relapse by 12 months [14–16]. Medication withdrawal is strongly recommended and its use can be viewed as a “reset” that then affords patients a greater likelihood of positively responding to appropriate prophylactics [13, 17, 18]. It is most helpful when patients are provided education and support about proper use of medications and taught strategies for avoiding relapse [19]. Studies have shown that such approaches can produce significant improvements that endure for extended periods, up to 5 years [20, 21].

CM-MO results in pervasive negative consequences, where personal suffering is accompanied by reduced quality of life and disability, and decreased abilities to participate in daily work and/or family activities, which often results in increasing symptoms of depression [12, 22–24]. Clinicians and researchers working in the field of headache disorders are becoming increasingly aware of the consequences of this condition, the resultant need for a multifactorial approach and treatment [25]. The joint use of pharmacological and non-pharmacological techniques has been shown to improve the health status of migraine patients and to enhance clinical outcomes by teaching and reinforcing patients to implement alternative procedures for addressing and coping with headache attacks [9, 26–30].

Among the wide array of available non-pharmacological treatments, mindfulness has been recently included in rehabilitation programs for chronic pain conditions [31–36]. Its efficacy has been addressed in a recent review of the psychological therapies in the neurorehabilitation of pain syndromes [37], where it has been judged as effective (Grade Level A) for chronic pain syndromes with heterogeneous physiopathology, exclusive of headache disorders. However when this review was prepared researchers had only begun to explore the utility of mindfulness for headache, so no firm recommendations could be made. The success with other pain conditions, however, has spurred researchers in the field of headache to increasingly turn their attention to mindfulness training as another viable alternative approach for supplementing patient care. The main goal of this approach is to increase patient awareness of their pain and improve their abilities to manage headache before resorting to their former medications [38–45]. As concluded in two recent reviews on the use of mindfulness-based approaches in headache disorders [46, 47], this kind of approach seems promising. In brief, literature findings [40–45] suggest that various mindfulness-based approaches may be helpful for headache sufferers, and that it may be of value also for those with CM-MO. However, the available studies are limited by an inadequate consideration of some of the most important endpoints in chronic headache research, namely the frequency of headache and the consumption of medications for acute headaches management. Further, other meaningful indicators of effectiveness, such as pain intensity, headache duration, disability, quality of life and some mental health-related variables, such as stress, anxiety, pain acceptance or self-efficacy, have yet to be fully explored. A second relevant shortcoming is the limited duration of follow-up reported in these studies, which has ranged between 3 weeks and 3 months. Finally, what is not clear is the ability of a mindfulness-based approach by *itself* to impact key primary as well as secondary migraine headache parameters, as well as promote reductions in consumption of acute medications. Two research areas, thus, warrant further attention: first, identifying optimal components and delivery schedules, by adequately specifying the intervention protocols; second, conducting rigorous controlled trials that assess the durability of effects over extended time periods, with appropriate control conditions and a clear specification of primary and secondary outcomes.

As a way to begin to address these uncertainties we conducted an exploratory clinical trial, one that compared conventional prophylactic pharmacological treatment *alone* to a mindfulness-based treatment *alone* for patients diagnosed with CM-MO and incorporated a more extended follow-up period. We carefully monitored the clinical course of these patients after all had undergone a structured withdrawal, with the hypothesis that the mindfulness-based approach would be similar in effectiveness when compared to conventional prophylactic treatment, for reducing headache frequency, consumption of acute medications, headache impact, symptoms of depression, and of anxiety.

## Methods

### Participants

Eligible patients were those diagnosed with CM-MO – i.e. code 1.3 and associated medication overuse, following the international criteria included in point 8.2 of the International Classification of Headache Disorder III edition, beta version (ICHD-3-beta) [5] – who presented consecutively for treatment at the Headache Centre of the Neurological Institute C. Besta of Milan, Italy, between February 2014 and June 2015. These patients were aged between 18 and 65 years and had a history of CM lasting for at least ten years that was associated with overuse of Triptans and non-steroidal anti-inflammatory drugs (NSAIDs) for a minimum of the past five years. Patients with comorbid major psychiatric disorders, namely psychotic disorders and personality disorders, determined on the basis of clinical history and psychiatric evaluation, or pregnancy were excluded.

### Procedures

All patients were first admitted to our out-patient day hospital service, where they participated in a 5-day structured medication withdrawal program that utilized intravenous therapy, including steroids and ademetionine [28, 48]. During withdrawal, patients were instructed to avoid the use of medications to manage any acute pain attacks. Upon completion of the structured medication withdrawal, all patients were encouraged to increase their physical activity and perform aerobic exercises, for 45 min twice per week, maintain a suitable level of hydration, and strive to consume 3 meals each day on a regular basis (emphasizing breakfast). Prior to discharge from the day treatment program, patients were informed of the possibility to participate in a new clinical trial, in which they could receive “medication alone” or “mindfulness training alone” (Med-Group or MT-Group).

Patients participating in the Med-Group received only prophylactic medications. The preventive compound was chosen on the basis of clinical history and medical comorbidities [49–52], such as done in routine care. Patients included in the MT-group, participated in a series of mindfulness training sessions and were not prescribed any form of prophylaxis. The mindfulness protocol we used was implemented on the basis of the Mindfulness-Based-Stress-Reduction MBSR program (MBSR) by Jon Zabat-Zinn [53]. Together with a close variant – the mindfulness-based cognitive therapy (MBCT) [54] – this is the most largely applied for various forms of recurrent pain [46], which we partially modified with regard to the frequency and the duration of sessions to increase the likelihood of adherence to treatment by patients. Training was provided in small groups (5–6 patients), that met in a relaxed and quiet room every consecutive Monday for 6 weekly sessions, each of about 45 min duration. All sessions were guided by an experienced neurologist trained in mindfulness practice. The order in which the different techniques and phases were administered– with the due caution and flexibility – was as follows. First, patients were provided a detailed explanation about the treatment protocol; i.e. what it is and what it is not, and in which clinical conditions it may be of most value. Second, patients were trained to assume a relaxed position that promoted good and regular breathing, while their eyes remained closed, with them maintaining a relaxed sitting position. Third, during the first meditations (approximately up to the second/third session), patients were invited to focus on attention on their breathing, on the present and on silence to enhance awareness of current mind and body sensations. Fourth, once patients learned to focus on the present, they erre requested to enhance awareness of their thoughts (third and fourth session), accepting them in a non-judgmental way. Fifth, in the last sessions (generally the last two), when patients had gathered higher awareness of their thoughts and the capacity to accept them, they were invited to preserve themselves from interfering thoughts, and to focus on the present and on the sensations they received from their bodies. When distractions occurred, patients were informed to resume attention to breathing and body awareness and observe the interfering content in a non-judgmental way [55]. Finally patients were encouraged to supplement their training with regular home self-practice, of 7–10 min per day (an amount typically recommended in studies of this type). The importance of practicing this form of meditation in an effortless manner was pointed out. Unlike prior investigations [41], we purposely limited the number of techniques to promote mastery of a few (versus exposure to numerous modalities where patients may be unable to master any to a meaningful degree) and keep time demands low and similar to that for pharmacological treatment.

This was an exploratory study, conducted in a working clinic that draws patients from a large coverage area, and the neurologist who guided the mindfulness sessions was one of the authors (LG), who had undergone extensive training in mindfulness at the Association for Meditation and Awareness under the supervision of Prof. Corrado Pensa. In this clinical setting it was not possible to implement random assignment, due to limited resources in terms of personnel and space. Another barrier lies in patients’ provenance and past history: in fact, our center is a high-level specialty one, and patients seeking treatment come from all over the country, with some of them being followed-up for many years, thus making it difficult to enroll patients in a way that is strictly consistent with the requirements of a RCT. Therefore, certain aspects were not blinded: in particular, the investigators knew which patients attended mindfulness sessions and which not, as those that did not attend the sessions needed to be supervised with regard to eventual side effects of prophylactic medications. Participation was, thus, on a voluntary basis, with patients self-selecting their preferred treatment condition. Patients opting for mindfulness training alone were informed of the importance of being available to attend weekly sessions on a consistent basis upon discharge. Patients unable to commit to the stated schedule were invited to participate in the medical prophylaxis alone condition. Follow-up evaluations were carried out by another neurologist (DD) to limit possible source of bias with clinical outcomes, and questionnaire completion was supervised by a psychologist (ES). Given these considerations we view this investigation as more along the lines of an *effectiveness trial* (versus an efficacy trial) [56]. The consequence of this is that our results have to be taken as preliminary and the efficacy has to be tested in future randomized trials.

The study was approved by the Institute’s Ethical Committee and written informed consent was obtained from each patient prior to enrollment in the study protocol. Follow-up assessments were conducted at 3, 6 and 12 months for all available patients. During this period, patients were instructed to continue their prior treatments. Patients in both groups were encouraged to restrict use of acute medications to headaches judged to be very disabling, operationally defined as a pain intensity rated as 8 or greater on a 0–10 (no pain – pain as bad as it could be). Patients were instructed to take Eletriptan (40 mg) and/or Almotriptan (12.5 mg) as the first-line treatment, and indomethacin (50 mg) as the second line; with regard to other NSAIDs, they were urged to take those medications that had already proved to be effective. Finally, in any case, they were strongly recommended to avoid opioids to the extent possible.

### Measures

Headache diaries [25], completed on a daily basis, provided the primary measure of outcome, i.e., headache frequency, and the consumption of acute medications (NSAIDs and triptans). The amount of single intakes was recorded, irrespective of the kind of medication.

The Headache Impact Test (HIT-6) [57] is a 6-item scale that measures lost time in 3 domains and other areas of impact (e.g., pain severity, fatigue, and mood), based on patient recall for the immediate past 4 weeks. Each item is rated on a scale ranging from “never” to “always.” Total scores range from 36 to 78 with higher scores indicating greater impact: scores ≥ 60 are indicative of a severe impact.

The Migraine Disability Assessment (MIDAS) [58] is the most widely used measure of disability in headache research and we included it to facilitate comparisons with prior research. It is composed of 7 questions, all referenced to the preceding 3 months. The first 5 inquire about the number of days during which headache presence disrupted (partially or totally) paid and school work, household work, and leisure/family/social duties. Summing these individual values yields a total disability score, which correspond to four severity level: 0–5, little or no disability; 6–10, mild disability; 11–20, moderate disability; 21 or above, severe disability. The remaining two items address the overall headache frequency and average pain intensity, measured on a 0–10 scale. As headache frequency and intensity were prospectively obtained from daily diaries, and the validity of headache data recalled over three months is questionable [59], we elected to not report these data here.

Beck Depression Inventory (BDI) [60]. We used the 13-item version, which asks participants to rate the extent to which they are experiencing each of the 13 common symptoms of depression included. Items are rated on a scale from 0 to 3 (where 3 represents the highest severity), with the maximum score being 39. When used as a screening device, a cut-off score of 9/10 seems best suited for indicating the presence/absence of depression [61].

The State-Trait Anxiety Inventory (STAI) Y1 and Y2 [62] is composed of two sections, each containing 20 items, that address state and trait anxiety, i.e., the transitory feelings that respondents experience in the moment in which they complete the questionnaire vs. the relatively stable and enduring personal features reflective of a predisposition to anxiety. Raw scores range between 20 and 80 for each scale, with higher scores indicating higher anxiety levels. The raw scores can be transformed into norm-based T-Scores (mean 50, SD 10) to enable comparability across gender and age groups [63].

### Data analyses

Mean baseline values for all demographic and dependent variables for the 2 treatment conditions were compared by *t*-tests. The primary and secondary outcome variables were analyzed in separate two-way mixed ANOVAs (Group: Mindfulness vs. Pharmacology x Time: Baseline, 3-, 6-, vs. 12-month follow-up), followed by post-hoc tests, with appropriate adjustments made when significant effects were obtained in order to guard against inflation of the familywise error rate. Partial eta squared (η^2^_*p*_) values were calculated for all significant findings, conservatively interpreting them as small (.01–.08), medium (.09–.24), and large (≥.25).

We additionally evaluated clinical significance by determining the percentage of patients that, compared to baseline evaluation, achieved a 50% or greater reduction in migraine frequency and the percentage of patients who no longer met the diagnostic criteria for CM at each of the three follow-up evaluations. We then compared the ratios between the two groups of patient (MT-Group and Med-Group), for each of these 2 additional measures, at each time-point using Chi-Squared analyses. The *p*-value for significance for all tests was set at .05.

## Results

Fifty patients met inclusion criteria during the study period, but six declined to participate due to lack of time or interest. Forty-four patients were therefore enrolled in this trial, 22 in each condition. In the Med-Group, five patients received valproate, eight botulinum toxin, five pizotifen, one amitriptyline, two received a combination of beta blockers and amitriptyline and one was given beta blockers and valproate. Table 1 reports the mean baseline values for all enrolled patients, collectively and by group assignment, along with current age and age at onset of headache. No differences were found between the 2 groups for any measures (the same was true when comparing baseline values for those who completed the trial; *n* = 39 versus those who did not, *n* = 3). At baseline, a similar percentage of patients were overusing triptans: 8 of 22 patients (36.4%) in the Med-Group and 6 of 22 patients (27.3%) in the MT-Group (χ ^2^(*1*) = 0.42, *p* = .52). The 2 groups did differ with respect to overuse of NSAIDS: 22 of 22 (100%) for Med-Group vs. 18 of 22 for MT-Group (χ ^2^(*1*) = 4.40, *p* = .04).

**Table 1:** Mean (and SD) baseline values for all measures for patients who began the trial, all 44 combined and separately for condition assignment, and statistical comparisons among the groups

Variable	Total (*N* = 44)		MT-Group (*N* = 22)		Med-Group (*N* = 22)		*t* (42)	*P*
	*M*	*SD*	*M*	*SD*	*M*	*SD*		
Age	44.5	9.2	45.6	9.3	43.5	9.2	0.75	.457
Age at Onset	20.4	9.0	21.5	10.5	19.3	7.3	0.80	.428
Headache frequency/month	20.5	7.9	19.2	7.8	21.9	7.8	-1.14	.263
Monthly medication intake	18.4	6.5	18.0	6.4	18.8	6.7	-0.41	.681
HIT	66.3	5.2	65.5	5.5	67.1	4.9	-1.05	.301
MIDAS	73.1	39.9	65.3	41.4	81.0	37.6	-1.32	.194
BDI-13	13.3	6.1	13.1	5.8	13.6	6.6	-0.32	.754
STAI-S	48.2	7.3	47.1	6.6	49.4	7.9	-1.06	.297
STAI-T	52.3	9.6	52.1	9.3	52.5	10.0	-0.13	.901

**Fig. 1:**
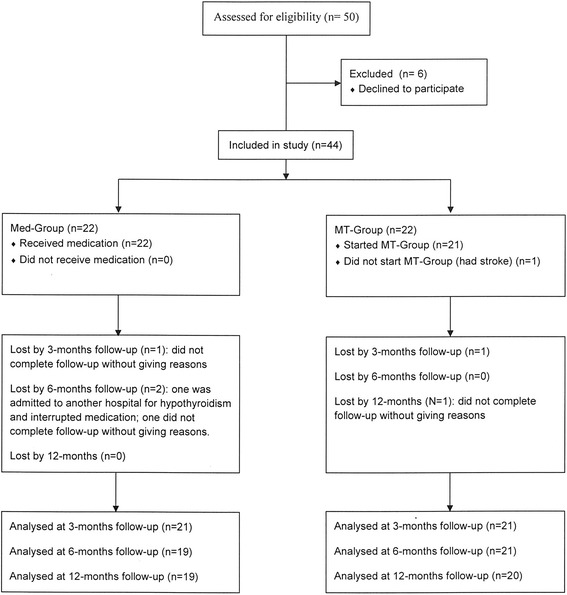
Flowchart of the study

Figure 1 shows the flow of patients in each of the two groups: complete follow-up data at 12 months was available for 19 patients in the Med-Group and for 20 in the MT-Group.

### Primary and secondary outcomes

**Fig. 2:**
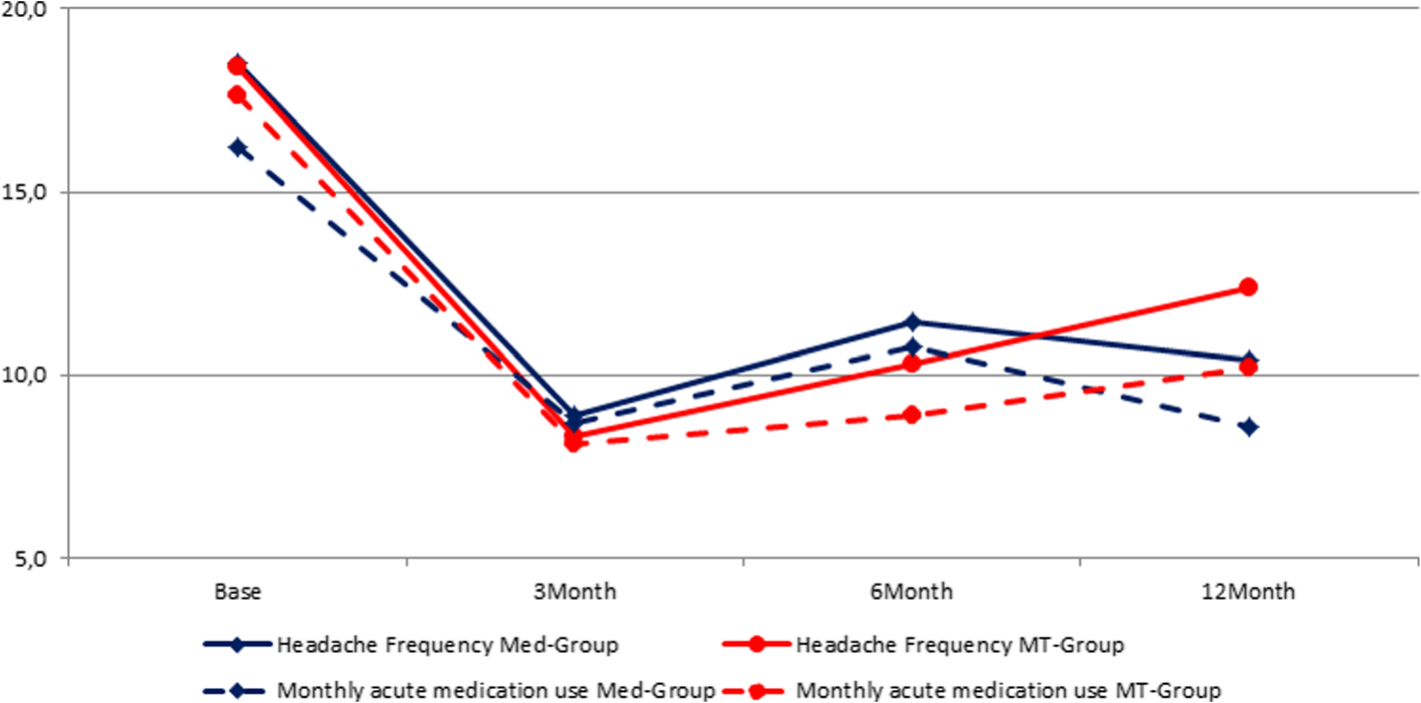
Longitudinal course of headache frequency and consumption of medication. *Note.* Differences were always significant compared to baseline; no differences were found between the Med-Group and the MT-Group at any time point

Mean values for all measures at all time points are reported for patients who completed the entire trial (see Table 2). Four of the seven separate mixed within-between subjects ANOVAs revealed significance only for the main effect of time— Headache Frequency, Medication intake, MIDAS, and BDI-13 (see Table 3). Analysis of the HIT-6 data revealed not only a main effect for time, but also for the interaction of time and group. Neither analysis for STAI-S or STAI-T revealed any significant effects (so these variables are not discussed further here). Pair wise post-hoc comparisons with Bonferroni corrections revealed all 3 follow-up points as significantly improved with respect to baseline values, but no differences among the 3 follow-up periods, for Headache Frequency, Medication intake, and BDI-13. MIDAS scores were significantly different from baseline for the 3- and 6-month follow-up, but not for the 12-month follow-up (see Table 4). Figure 2 graphically presents the outcomes for headache frequency and consumption of medication for management of acute headaches over the 12-month follow-up period.

**Table 2:** Mean values and SD’s for mindfulness (MT-Group) and pharmacology (MED-Group) at each measurement period

	MT-Group				Med-Group			
	Baseline	3-MO	6-MO	12-MO	Baseline	3-MO	6-MO	12-MO
Headaches Frequency	18.5 ± 7.2	8.3 ± 3.5	10.4 ± 6.9	12.4 ± 8.5	18.5 ± 7.2	8.9 ± 8.0	11.4 ± 8.0	10.4 ± 7.2
Medications intake	17.7 ± 5.9	8.1 ± 4.6	8.9 ± 4.2	10.3 ± 5.4	15.4 ± 4.4	8.8 ± 8.4	11.0 ± 7.6	8.6 ± 4.8
HIT-6	65.3 ± 5.7	62.0 ± 5.7	60.7 ± 10.8	64.5 ± 7.0	66.9 ± 5.2	60.7 ± 7.7	62.6 ± 6.3	61.5 ± 4.8
MIDAS	65.4 ± 43.5	39.0 ± 36.7	41.5 ± 51.7	53.7 ± 52.6	82.9 ± 40.0	26.7 ± 23.5	38.8 ± 25.4	51.5 ± 50.2
BDI-13	13.4 ± 5.9	9.0 ± 6.3	9.0 ± 5.3	10.3 ± 6.8	13.3 ± 6.8	6.2 ± 6.3	8.0 ± 5.9	7.6 ± 6.4
STAI-T	52.3 ± 9.8	48.6 ± 8.4	48.6 ± 7.7	50.9 ± 9.5	52.8 ± 10.2	48.5 ± 9.4	51.3 ± 9.9	48.4 ± 9.6
STAI-S	47.0 ± 6.8	45.4 ± 6.4	45.5 ± 6.9	49.9 ± 9.3	49.6 ± 7.8	47.2 ± 6.1	48.3 ± 10.7	48.6 ± 8.7

*N* = 19 for MT-Group and *N* = 20 for MED-Group

*Note.* Values are expressed as means ± SD

**Table 3:** Mixed within-between ANOVA results

Variable	Main effects for time			Main effect for group		Interaction (Time X Group)		
	Wilks’ lambda	*P*	partial η^2^	*F*	*p*	Wilks' lambda	*p*	partial η^2^
Headaches frequency	.43	< .001	.57	0.00	.959	.93	.453	.07
Medications intake	.31	< .001	.69	0.04	.842	.83	.094	.17
HIT	.67	.002	.34	0.02	.902	.76	.020	.24
MIDAS	.43	< .001	.57	0.02	.902	.86	.141	.14
BDI-13	.49	< .001	.51	1.12	.297	.93	.475	.07
STAI-S	.82	.064	.19	0.70	.408	.93	.481	.07
STAI-T	.82	.068	.18	0.00	.952	.89	.246	.11

**Table 4:** Post-hoc comparisons across time for significant time main effects

Variable	*M*	*SD*	*M* diff	*P*
Freq				
Baseline (ref)	18.5	7.1		
3-month	8.6	6.1	9.9	< .001
6-month	10.9	7.4	7.6	< .001
12-month	11.4	7.9	7.1	< .001
Medications intake				
Baseline (ref)	16.6	5.3		
3-month	8.4	6.6	8.2	< .001
6-month	9.9	6.1	6.7	< .001
12-month	9.5	5.1	7.1	< .001
MIDAS				
Baseline (ref)	73.9	42.2		
3-month	33.0	31.2	40.9	< .001
6-month	40.2	40.6	33.7	< .001
12-month	52.6	50.8	21.3	ns
BDI-13				
Baseline (ref)	13.3	6.2		
3-month	7.6	6.4	5.7	< .001
6-month	8.5	5.5	4.9	< .001
12-month	9.0	6.7	4.4	.001

*Note*: Only comparisons to baseline scores are presented in this table, as all other pairwise comparisons were not statistically significant

**Fig. 3:**
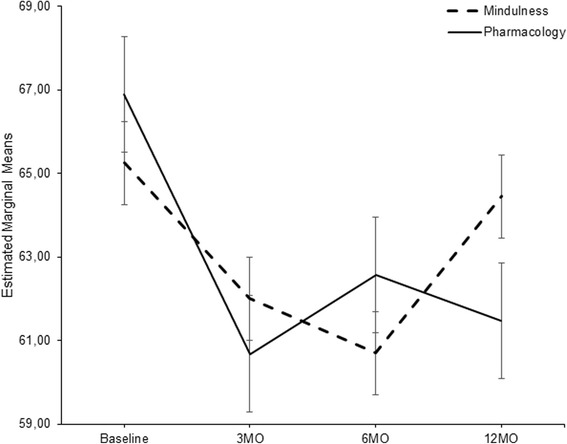
Estimated marginal means for HIT-6 scores across time for Mindfulness and Pharmacology groups. Error bars represent standard errors

To investigate the source of the significant interaction effect for the HIT-6, we conducted all pairwise comparisons for all time points for each group separately (see Table 5 and Fig. 3). Pairwise comparisons, with Sidak correction, revealed no differences between any time points for the MF-Group. For the MED-Group the 3-month and 12-month values were significantly reduced when compared to baseline. No other differences emerged.

**Table 5:** Pairwise comparisons between time points of HIT for each treatment group

Group	Comparisons		Mean difference (A-B)	*SE*	*p*	95% CI of difference	
	Time A	Time B				*LL*	*UL*
Mindfulness	Baseline	3 M	3.25	1.56	.240	-1.10	7.60
		6 M	4.55	2.21	.247	-1.58	10.68
		12 M	0.80	1.73	.998	-4.00	5.60
	3 M	6 M	1.30	1.87	.983	-3.91	6.51
		12 M	-2.45	1.81	.705	-7.48	2.58
	6 M	12 M	-3.75	1.43	.073	-7.73	0.23
Pharmacological	Baseline	3 M	6.21	1.60	.003	1.75	10.67
		6 M	4.32	2.26	.328	-1.97	10.60
		12 M	5.42	1.77	.024	0.50	10.34
	3 M	6 M	-1.90	1.92	.910	-7.24	3.45
		12 M	-0.79	1.86	.999	-5.95	4.37
	6 M	12 M	1.11	1.47	.974	-2.97	5.18

*Note.* Multiple comparisons were adjusted with Sidak correction

### Percent improvement and percent of patients no longer meeting criteria for CM

Table 6 reports the percentage of patients showing 50% or more reduction of headaches compared to baseline and of patients no longer meeting criteria for CM for each of the time-points. For both of the clinical endpoints, there were no differences between patients in the MT-Group and those in the Med-Group. With regard to the percent improvement variable, the trend had a U-shaped curve in both groups, while the trend had a J-shaped curve with regard to the number of patients no longer meeting the criteria for CM.

**Table 6:** Percent improvement and percent of patients no longer meeting criteria for CM at each time-point

		MT-Group	Med-Group	Chi-Squared (P-value)
50% reduction	3 Months	57.1%	76.2%	1.71 (*P* = .190)
	6 Months	47.6%	26.3%	1.93 (*P* = .165)
	12 Months	50.0%	52.6%	0.03 (*P* = .869)
No longer CM	3 Months	95.2%	90.0%	0.41 (*P* = .520)
	6 Months	76.2%	78.9%	0.04 (*P* = .835)
	12 Months	65.0%	73.7%	0.35 (*P* = .557)

## Discussion

Our preliminary data show that both groups of patients, treated with only a single, non-combination intervention—conventional pharmacological approach only versus a mindfulness-based approach only—revealed significant decreases in number of monthly headache days, monthly consumption of medication for acute headache management, MIDAS, and depressive symptoms up to 12-months follow-up. The change in mean BDI-13 scores (collapsing across groups), appears to be of clinical significance, as the baseline values, which fell into the stringent range (13/14) for tentatively identifying moderate to severe clinical depression, had by 12 months fallen overall within the lowest end of the range suggesting the possible presence of moderate/severe depression (9/10). Although MIDAS scores decreased from baseline by 28.8% at 12 months, the mean score at this time point (averaged across both conditions) continued to fall within the highest severity grade Level (IV). Headache impact was reduced to a statistically significant degree at 3- and 12-month follow-up, but only for the MED-Group. It is important to point out that at all time points, neither group revealed clinically meaningful reductions on this measure because means continued to fall within the highest severity category for this scale (all means ≥ 60). Given the long-standing duration of chronic headache activity by our patients, it is not surprising that headache impact and depression did not reveal more marked changes, even at 1 year follow-up. Changes of a psychological nature often take additional time to fully manifest [26]. Anxiety scores remained unchanged over time. Although anxiety disorders and CM are known to be comorbid [64] – STAI scores revealed the absence of significant anxiety problems in either group, thus leaving little room for change (“basement” or floor effect).

The proportion of patients achieving a 50% reduction in headaches frequency (a measure commonly used to evaluate “clinically significant improvements”) was similar at all time-points (at 12 months: 50% in the MT-Group, 52.6% in the Med-Group), and the same was true for the proportion of patients no longer meeting the CM criteria (at 12 months: 65% in the MT-Group, 73.7% in the Med-Group). Taken as a whole, our results suggest that the longitudinal course of patients receiving Mindfulness-based treatment, and who were instructed to refrain from medical prophylaxis (which was verified by the dairy records participants maintained throughout the study), was overall very similar to that for patients who were administered conventional medical prophylaxis, with few exceptions noted.

Our preliminary data extend the findings of Wells and colleagues [40], whose mindfulness intervention was focused on episodic migraine and reported more limited findings. To our knowledge, only one previous study has examined the utility of mindfulness-based treatments for chronic forms of headache (CM or Tension-Type Headache, with the distribution not being reported) wherein mindfulness was also examined as an “add-on” therapy and consisted of a host of other therapeutic components, some derived from a mindfulness framework but many derived from other theoretical models [41]. These investigators found significant differences between MBSR + pharmacoterapy and pharmacotherapy alone with respect to perceived pain intensity and quality of life. However, it is not clear if their treatment impacted headache frequency, which is our primary measure and the primary outcome recommended by the most recent IHS clinical trial guidelines [65], or other outcome measures such as those that we included, i.e., use of acute medications, disability burden and mood. The fact that positive effects (although not always reaching significant changes) were observed in our study for these varied measures in patients who received our brief mindfulness training alone (in the absence of prophylactic medications) suggests that mindfulness-based treatment may be comparable to standard pharmacological prophylaxis as far as its global positive clinical improvement. However, the absence of random assignment and the fact that our study was not cast as a non-inferiority trial leaves this possibility more as a hypothesis, one in need of further testing.

Mood is one of the most important non-headache factors associated with migraine chronification [7, 64] and reducing headache frequency can lead to reductions in depression [29, 66]. Nonetheless, the relevance of depressive symptoms remains somewhat controversial. In fact, in previous studies on CM-MO samples [24, 67] depression scores – measured with the updated BDI-II [68] and not with the original BDI – were not correlated with frequency of headaches and, when implemented in a predictive model together with headache frequency and pain intensity, BDI-II scores had higher value in predicting disability and quality of life scores. Our finding that Mindfulness practice (as well as medication) had a modest positive effect on levels of reported depression over time is compatible with a conclusion previously drawn in studies addressing depression and mindfulness-based treatments [69–73]. These findings show that the effect of mindfulness-based approaches on symptoms of depression were superior to psycho-educational intervention and non-inferior to individual cognitive behavioral therapy, that they yielded similar results compared to antidepressant therapies and, finally, that the effect is maximized when the treatment is combined. Considering that only 40% of patients in the MED-group received a therapy having some kind of mood-modulating effect, the finding that the impact on mood component was similar is in line with the previous report, and suggests that mindfulness-based treatments, combined with appropriate antidepressant therapy, might yield an increased impact on symptoms of depression.

Although mean BDI values for both groups were in the range of a significant levels of depression prior to treatment, we hesitate to speculate further about the meaningfulness of the changes reported here given that our measure of depression is intended primarily as a screening instrument and does not take the place of a careful clinical diagnosis.

More recent conceptions, wherein migraine is being recognized as a condition in which biological, social and psychological aspects are very interconnected [25] has helped to increase awareness of the need to modify therapeutic approaches to include newer and sometimes “non-conventional” options (behaviorally and cognitively based), along with “traditional” treatments (i.e., medical) to better help patients to manage their condition, reduce their medication intake, and minimize the incidences of relapse in overuse after withdrawal [25, 74].

Mindfulness is designed to promote the ability to focus on and accept the present situations and the difficulties of every day. As demonstrated by Kabat-Zinn [75], patients who have been educated to use mindfulness may better manage stressful situations, increase their self-efficacy, and learn to manage pain more adequately avoiding the compulsion between pain and medication intake which easily sets in motion the vicious cycle of pain and medication and to the condition of overuse. Mindfulness research, especially as regards headache, remains in the infancy stage, with many aspects in need of further investigation. Among these are determining which of the myriad of bio-psycho-social factors may underlie treatment effectiveness, including, for example, changes in perception of and reactions to pain sensations and emotion, self-efficacy and coping abilities, physiology, cerebral structures and circuits [19, 38, 46, 47].

Although our findings are encouraging and suggestive of the independent value of mindfulness for headache care, certain design limitations preclude us making unequivocal claims. Our inability to randomize patients to conditions serves as a limiting factor, with results perhaps applying only to those particularly motivated to commit to an extended training period for mindfulness. Our headache center is designed primarily as a fee for service clinic, where patient preferences must be considered. However, as pointed out by Nash et al [56] trials of this type, more aptly termed “effectiveness trials” (versus the more standard “efficacy trials”) clearly have a place in the early stage of treatment development. In this case, we hope our findings serve to expand recently published data on patients with primary headaches supporting the clinical value of mindfulness in the most severely affected patients in the migraine spectrum; i.e., those with chronic migraine coupled with medication overuse. Another consideration is our inability to document the extent to which patients adhered to each treatment (no dose monitoring for the MED group and no checks for amount of mindfulness practice or the depth of learning patients acquired). Nonetheless, we believe our findings support the value of conducting further more well-controlled studies (incorporating random assignment, larger samples sizes, and checks on integrity of treatment) are warranted to more fully explore the benefits, boundaries, and mechanisms of action for mindfulness in treating chronic migraine by itself and when it is complicated by medication overuse and medical or psychological comorbidities. Finally, our sample was composed of patients with CM and with no psychiatric comorbidities. Two literature reviews showed that migraineurs, compared to the general population, are much more likely to suffer from psychiatric comorbidities (up to 60-70% for mood and anxiety disorders) and that women with migraine with aura are at an increased risk of suicide attempt [76, 77]. Conversely, the evidence on the relationships between mood disorders expression and suicidal ideation seems contrasting, with some studies finding and others not finding any connections [78, 79]. Patients included in our sample seem to be clearly different from those described in these previous studies, as they did not have psychiatric comorbidity – although some degree of low mood was found – and actually we had no reasonable ground to suspect any suicidal ideation among the participants herein included. Caution is therefore recommended before generalizing our results to the entire population of CM patients: for all of the above reasons, our results should be taken as preliminary.

## Conclusions

Our results provide initial support for the beneficial effect of Mindfulness-based treatment in the management of chronic migraine that is accompanied with medication overuse, a headache form which represents a clinical challenge. Our results further suggest that a Mindfulness-based treatment may be comparable to standard pharmacological prophylaxis with regard to relevant primary outcomes such as headaches frequency reduction and reduction in the consumption of acute medications.

## Abbreviations

BDI: Beck depression inventory
CM: Chronic migraine
CM-MO: Chronic migraine associated with medication overuse
HIT-6: Headache impact test
ICHD-3Beta: International Classification of Headache Disorder III edition, beta version
MBCT: Mindfulness-based cognitive therapy
MBSR: Mindfulness based stress reduction
Med-Group: Medication alone group
MIDAS: Migraine disability assessment
MO: Medication overuse
MT-Group: Mindfulness training alone group
NSAIDs: Non-steroidal anti-inflammatory drugs
STAI: State-trait anxiety inventory
TAU: Therapy as usual
TTH: Tension-type headache
